# Ophthalmologic Psychophysical Tests Support OCT Findings in Mild Alzheimer's Disease

**DOI:** 10.1155/2015/736949

**Published:** 2015-05-27

**Authors:** Elena Salobrar-Garcia, Rosa de Hoz, Blanca Rojas, Ana I. Ramirez, Juan J. Salazar, Raquel Yubero, Pedro Gil, Alberto Triviño, José M. Ramirez

**Affiliations:** ^1^Instituto de Investigaciones Oftalmológicas Ramón Castroviejo, Universidad Complutense de Madrid, Madrid, Spain; ^2^Facultad de Óptica, Universidad Complutense de Madrid, Madrid, Spain; ^3^Facultad de Medicina, Universidad Complutense de Madrid, Madrid, Spain; ^4^Hospital Clínico San Carlos, Madrid, Spain

## Abstract

*Purpose*. To analyze in mild Alzheimer's disease (MAD) patients, GDS-4 (Reisberg Scale), whether or not some psychophysical tests (PTs) support OCT macular findings in the same group of MAD patients reported previously. *Methods*. Twenty-three MAD patients and 28 age-matched control subjects with mean Mini Mental State Examination of 23.3 and 28.2, respectively, with no ocular disease or systemic disorders affecting vision were included. Best-corrected visual acuity (VA), contrast sensitivity (CS) (3, 6, 12, and 18 cpds), color perception (CP), and perception digital test (PDT) were tested in one eye of each patient. *Results*. In comparison with the controls, MAD patients presented (i) a significant decrease in VA, PDT, and CS for all spatial frequencies analyzed, especially the higher ones, and (ii) a significant increase in unspecific errors on the blue axis (*P* < 0.05 in all instances). In MAD patients, a wide aROC curve was plotted in all PTs. *Conclusions*. In MAD, CS, VA, and the tritan axis in CP were impaired. The PTs with the greatest predictive value are the higher spatial frequencies in CS and tritan unspecific errors in CP. PT abnormalities are consistent with the structural findings reported in the same MAD patients using OCT.

## 1. Introduction

Alzheimer's disease (AD) is a neurodegenerative disorder of the central nervous system characterized by cortical atrophy most pronounced in the medial temporal and posterior temporoparietal regions [1]. Disturbances of short-term memory, judgment, and emotion are characteristic of the disease.

As an extension of the central nervous system, the retina displays similarities to the brain in terms of anatomy, functionality, response to insult, and immunology [2–4]. Furthermore, the retina presents manifestations of major neurodegenerative diseases, and it has been argued that several ocular diseases should be viewed as forms of neurodegenerative disorders [2]. This unique property makes the retina a valuable tool for* in vivo* visualization and study of retinal changes.

The first histopathologic evidence of postmortem retinal changes from AD patients was reported by Hinton et al., who found a significant axonal degeneration in the optic head nerve [5]. Further studies have demonstrated that a decrease in retinal ganglion cell numbers was associated with an array of intracellular injuries [6]. The analysis on the distribution of neuronal loss in the retina by Blanks et al. showed that the most pronounced areas of cell loss in the ganglion cell layer (GCL) were the superior and inferior retinal quadrants [7] and there was an extensive loss of RGC in the temporal foveal region [8]. These findings have been corroborated using the Optical Coherence Tomography (OCT), demonstrating the decrease of the RNFL and the macular thickness [9–16], even in early stages of mild cognitive impairment [12, 17–19].

Currently it is thought that RGC loss in AD might result from amyloid pathology in the retina. Both amyloid-beta plaques and oligomers have been reported in the human retina [4, 20–25]. Therefore, amyloid accumulation in the retina of patients with AD may result in the degeneration of RGCs in parallel with amyloid-beta-related neurodegeneration in the brain [25]. However, retinal findings in AD could be also due to retrograde degeneration of RGCs secondary to AD pathology in the central visual pathways, given that AD patients show degeneration of the primary and secondary visual cortex [26].

In addition to the anatomical findings in AD patients, this disease can exert an impact on most aspects of visual processing, such as visual field abnormalities [27–29], color perception deficits [30–32], pattern electroretinogram changes [11, 33, 34], and reduced contrast sensitivity (CS) [35–38].

The M visual pathway appears to be particularly vulnerable to degeneration in AD [26]. Thus, degeneration of RGCs and/or visual pathways in the brain may underlie the functional deficits in CS seen in patients with AD [29]. CS function in humans diminishes with changes in the retina, the optic nerve, and lesions of the occipital, temporal, and/or parietal cortices [39].

Psychophysical investigations of CS in AD patients have rendered results consistent with the neuropathological evidence. However, studies of CS in patients with AD have yielded variable results. Some studies have reported no AD-related deficits in spatial CS [40, 41], while others have found deficits at all spatial frequencies tested [42].

OCT macular studies in AD by our group and others have recently reported that mild AD (MAD) patients with a high mean score (23.3 ± 3.1) on the Mini Mental State Examination (MMSE) had significantly reduced macular nerve fiber layer thickness with or without significant peripapillary involvement [9, 43, 44]. In the present study, to analyze whether these anatomical features coexist with alterations in the visual functional state in MAD patients, we include the same patients from the previous work [9]. To examine the visual pathway in this homogeneous group (in terms of disease stage) of MAD, we performed different psychophysical tests, specifically visual acuity (VA), CS, color perception, and perception digital test (PDT). The results are compared with those of an age-matched control group in order to (i) improve our understanding on how the visual pathway works in MAD and (ii) determine whether or not these tests support early macular findings on OCT in MAD.

## 2. Materials and Methods

### 2.1. Subjects

To select patients, we reviewed the Database of the Memory Unit of the Hospital Clínico San Carlos in Madrid (Spain), consisting of a total of 2635 patients. First, we excluded the patients with a Global Deterioration Scale (GDS) over 4 [45] and then those with a mood or psychiatric disorder. Next, we took into account 87 patients with MAD. These patients, according to the National Institute of Neurological and Communicative Disorders and Stroke-Alzheimer's Disease and Related Disorders Association and the Diagnostic and Statistical Manual of Mental Disorders IV, had mild cognitive impairment according to the Clinical Dementia Rating Scale. Then ophthalmic medical records of these patients were reviewed, excluding those patients who were previously diagnosed with an ophthalmic condition. After this analysis, 29 patients with AD had all the requirements to participate in the study (GDS over 4 and freedom of ocular disease and systemic disorders affecting vision in their medical record). Of the 29 MAD patients and 37 age-matched control subjects selected (normal MMSE scores), 6 MAD patients and 9 age-matched control subjects were subsequently excluded due to posterior pole pathology including macular degeneration, drusen, suspicion of glaucoma, glaucoma, epiretinal membrane, or cataract that prevented ocular examination. Because of this selection, 23 patients with MAD and 28 age-matched control subjects were considered for the study. Informed consent was obtained from both groups. The research followed the tenets of the Declaration of Helsinki, and the protocol was approved by the local ethics committee.

### 2.2. Methods

The clinical evaluation of our MAD included a review of the medical records, a caregiver interview, physical and neurological examinations, a psychometric test, neuroimaging techniques, and routine laboratory testing for dementia.

For the ophthalmological part of the study only one eye of each patient was analyzed. All participants met the following inclusion criteria: being free of ocular disease, AREDS Clinical Lens Standards <2, retinal drusen and systemic disorders affecting vision, having a best-corrected VA of 20/40, having a ±5 spherocylindrical refractive error, and having intraocular pressure of less than 20 mmHg. For screening, all AD patients and control subjects underwent a complete ophthalmologic examination, including assessment of VA, refraction, anterior segment biomicroscopy, applanation tonometry (Perkins MKII tonometer, Haag Streit, Reliance Medical, Switzerland), CS test CSV-1000E (VectorVision, Greenville, OH, USA), Roth 28-hue color test (Luneau, Paris, France), PDT [46], and dilated fundus examination. In the dilated fundus examination, no differences were found between MAD patients and age-matched control subjects. These tests were selected considering that in this developmental stage of the disease the results were not influenced by the patient's cognitive impairment.

### 2.3. Visual Acuity

Monocular best-corrected distant VA was determined using a standard clinical Snellen eye chart. The correction was based on the subjective refraction of the subject. The patients started to read each row from the top of the Snellen eye chart and proceeded toward the bottom. This was ended when the hit rate was less than five of eight (an approximation to 56.25%, the steepest point of the psychometric acuity function).

### 2.4. Contrast Sensitivity Function

The CS test was performed under the same conditions for all the patients and with the CSV-1000E system (VectorVision, Greenville, OH, USA) and in the presence of best-corrected VA for far vision. The CSV-1000E test provides a fluorescent luminance source that retroilluminates a translucent chart and is able to monitor and autocalibrate the light level to 85 cd/m^2^. The CSV-1000E was performed at 98.5 inches as recommended by the manufacturer. The nonselected eye was occluded for each measurement. The translucent chart presents four spatial frequencies: 3, 6, 12, and 18 cyc/deg. Each spatial frequency was presented on a separate row of the test. Each row presented 17 circular patches 1.5 inches in diameter. The first patch in the row had a very high contrast grating (sample patch) on the far-left side of the row. The remaining 16 patches appeared in 8 columns presented across the row. In each column, one patch presented a grating while the other patch remained blank. The patches that presented gratings decreased in contrast from left to right across the row. The patient was directed to observe the first sample patch and to look for the grating pattern in each column. While reading across the row, the patient indicated whether the grating appeared in the top patch or the bottom patch of each column. If the grating was not visible in either patch, the patient was to report that both were blank. The patient was encouraged to guess whether a grating was at least partially visible as the threshold was approached. However, the patient was cautioned that if no gratings were visible, then the response should be “both blank.” The contrast level of the last correct response was recorded as the threshold.

### 2.5. Color Perception

To administer a color-vision test that did not require a naming response, we used Roth 28-hue color test (Luneau, Paris), a quick and easy color arrangement test first described by Roth [47]. Color perception was assessed in the presence of best-corrected VA for near vision. The test uses the equivalent of every third color cap from the Farnsworth-Munsell 100 (F-M 100) as an abbreviated version. Subjects were instructed to select the cap most similar to the reference cap, then the cap most similar to the previously chosen one, and so on and to place them in sequential order until all 27 caps were arranged in a circular sequence. Test instructions were repeated by the examiner during test when necessary. The time to perform the test was not restricted and the subject was allowed to make corrections. The results were recorded on diagrams provided by the manufacturer which depict the direction of axes corresponding to several types of color-vision defects. Errors classified the observer as protanomalous, deuteranomalous, or tritanomalous (red-, green-, or blue-deficient, resp.). Following the manufacturer's manual [48] blue axis errors were considered when caps 43 to 64 were malpositioned. In this way, the tritan errors were quantified.

### 2.6. Perception Digital Test (PDT)

The PDT, developed in 2007 [46], is an easy, fast, and sensitive method for evaluating disorders of visual perception in MAD patients. The aim of the test is to assess the visual recognition of familiar situations, masked by geometric special effects that hinder perception. The test includes 15 sheets. Each sheet shows the same picture at different positions in space. The pictures are distorted by the choice of special effects: geometric effect (tile) and effect of the frame 24/48 of MGI Photo Suite III program. The test includes five photographs of landscapes, six of common objects, two of people, one of an animal, and one of a letter. The patient had to identify the picture that was properly oriented in space.

### 2.7. Statistical Analysis

Data for the statistical analysis were introduced and processed in SPSS 19.0 (SPSS Inc.©, Inc., Chicago, IL, USA). The data are reported as mean values ± SD. The differences between MAD and control eyes were analyzed using the Mann-Whitney test. Sensitivity at 90% specificity and area under the receiver operator characteristic (aROC) analysis for discriminating between healthy and MAD patients were calculated for all the psychophysical tests analyzed. The association between the tests and MMSE was evaluated by Pearson's correlation coefficient. A *P* value of <0.05 was considered statistically significant.

## 3. Results

Demographic and clinical data for the MAD patients and control group are shown in Table 1. There were no statistically significant differences in age, gender, or educational level between the study groups. The MMSE scores in MAD patients were significantly decreased in comparison with age-matched control subjects (Table 1). All MAD patients had MMSE values higher than 17.

### 3.1. Visual Acuity

**Figure 1:**
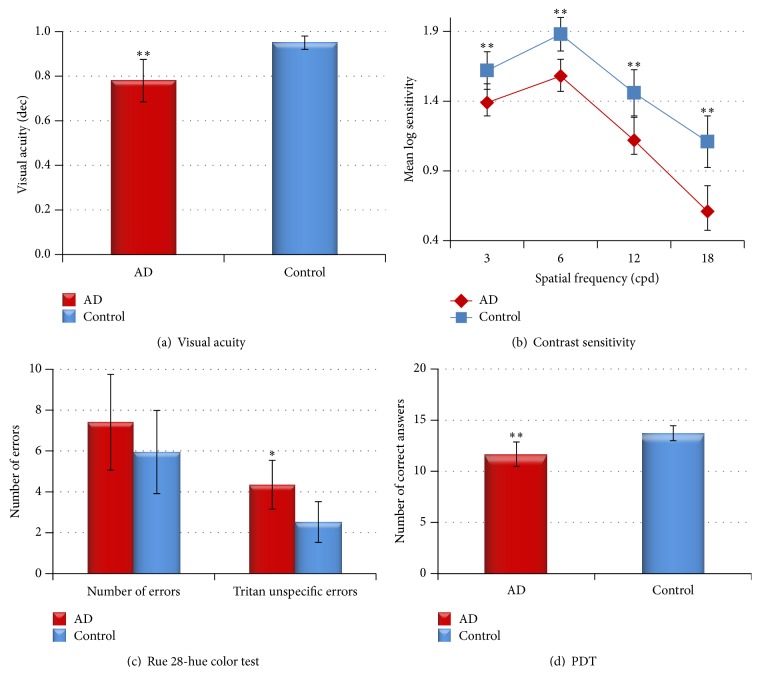
Mean data of the psychophysical tests. (a) Visual acuity, (b) contrast sensitivity, (c) desaturated Rue 28-hue color test, and (d) perception digital test. Each bar represents the mean ± SD. ^*∗*^ *P* < 0.05 versus control. ^*∗∗*^ *P* < 0.01 versus control. Mann-Whitney *U* test.

**Figure 2:**
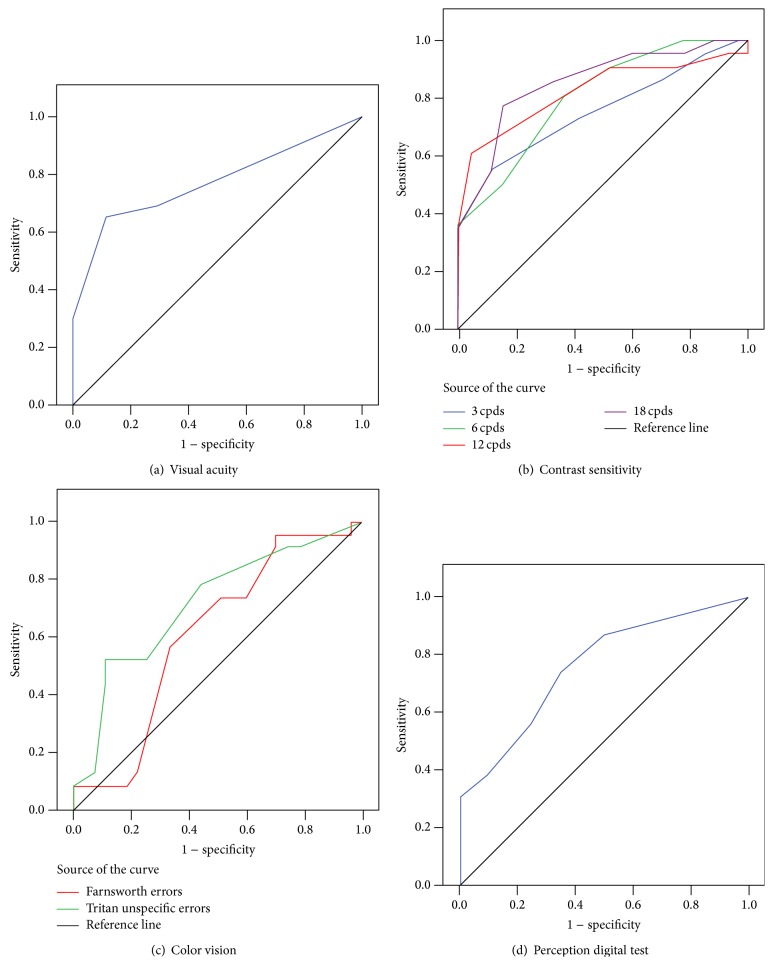
Areas under the ROC curves of the psychophysical tests in discriminating between mild AD patients and control subjects. (a) Visual acuity (dec), (b) contrast sensitivity, (c) Rue 28-hue color test, and (d) perception digital test.

The mean VA in MAD patients significantly decreased in comparison with the age-matched control group (Table 2; Figure 1(a)). VA showed a wide aROC (Table 3; Figure 2(a)). A positive and statistically significant linear association was found between VA and MMSE score (Table 4).

### 3.2. Contrast Sensitivity

All analyses were conducted with log CS values. 30.43% of the MAD patients (*n* = 7) were not able to report the orientation of the 18-cpd grating at the highest contrast level. Three of these 7 MAD patients were also unable to detect the 12-cpd grating at any contrast value. The analysis of CS of the MAD patients revealed a statistically significant reduction at all spatial frequencies tested (3, 6, 12, and 18 cpds) in comparison with age-matched control subjects (Table 2; Figure 1(b)). In addition, it was found that the higher the spatial frequency, the greater the loss of CS perception. Thus, in MAD patients the spatial frequency of 18 cpds showed the greatest decrease in CS (44.57%) compared with the age-matched control group. The analysis of the ROC curves (Table 3; Figure 2(b)) showed that, for MAD patients, the highest spatial frequencies analyzed (18 and 12 cpds) had the widest areas under the ROC curves for all the parameters analyzed. The 18-cpd frequency had the strongest correlation, followed by the 12-cpd frequency. A good correlation was detected also for the 6- and 3-cpd frequencies (Table 3; Figure 2(b)).

A positive and statistically significant linear association was found between CS for all spatial frequencies tested and MMSE score (Table 4).

### 3.3. Color Perception

The analysis of color vision with the Rue 28-hue test showed that there were no significant differences in the color perception between patients with MAD and age-matched control group based on the total error score (Table 2). However, the analysis of the tritan axis revealed that (i) the number of tritan unspecific errors was significantly increased in MAD patients in comparison with age-matched control (Table 2; Figure 1(c)), (ii) aROC curve drawn for the total tritan unspecific errors was statistically significant (Table 3; Figure 2(c)), and (iii) there was a negative and statistically significant linear association between tritan unspecific errors and the MMSE score (Table 4).

### 3.4. Perception Digital Test (PDT)

The PDT mean value found for the control group was significantly higher than that of the MAD group. The analysis of the individual sheets revealed a significant difference in sheets 2, 8, and 10 between MAD and age-matched control subjects (Table 2; Figure 1(d)).

The aROC curve for PDT showed a statistically significant wide area. However, when the analysis was calculated by individual sheets, no significant differences were found (Table 3; Figure 2(d)). A positive and statistically significant linear association was found between the PDT and MMSE score (Table 4).

## 4. Discussion

AD patients reportedly manifest subjective visual complaints, including the inability to read, spatial deficits, or difficulty in recognizing faces despite having relatively good visual acuity values and visual fields. For this reason the patients in our study were carefully selected so that sample was quite uniform regarding VA and MMSE. In addition, between different SC tests available, the CSV-1000 was chosen because results are more independent with respect to the VA [37].

One of the relevant points of the present study concerns MMSE value. Our MAD patients were homogeneous in terms of their disease stage and had a high MMSE mean value (23.3 ± 3.1), higher than any value found in the literature [36, 37, 49–51]. This means that all MAD patients included in this study were in a very early stage of the disease.

### 4.1. Visual Acuity

VA values in the present study were within normal limits for this age range. However, mean VA in MAD patients was significantly decreased in comparison with age-matched control subjects. VA values in AD patients have been controversial. Thus, several studies reported no decrease in VA in patients with Alzheimer's neurodegeneration [36, 40, 41, 50, 52–54] while others showed a decrease in VA [55] which in some instances was associated with visual hallucinations [56].

### 4.2. Contrast Sensitivity

We found that, in comparison with age-matched control subjects, CS in MAD patients was significantly reduced in all spatial frequencies, the reduction being more pronounced at higher frequencies. Specifically, at a spatial frequency of 18 cpds, MAD patients showed a 44.57% CS reduction in comparison with age-matched control. High spatial frequencies are recorded in the P cells of the retina, which are more concentrated in the macular area. Low spatial frequencies are recorded by the M cells located over the entire retina [57]. Our CS results suggest that MAD patients undergo an impairment of both P- and M-cell function, P cells being the most affected. This finding correlates with data recently published by our group [9] in which patients with MAD had a significant decrease in the nerve fiber layer thickness in the macular area compared with age-matched controls. By contrast, the significant decrease in CS for the lower spatial frequencies found here in MAD patients did not parallel OCT findings in the peripapillary region in the fact that the decrease in the nerve fiber layer thickness reported by our group in this retinal region did not reach significance when compared with controls. These findings tempt us to postulate that, in MAD, CS seems to detect the M pathway impairment earlier than OCT does.

CS in AD has been analyzed for several years. Although in two studies no differences between AD and age-matched control were detected [40, 54], most reports found that CS function is impaired in AD patients. In some instances, CS was reduced at all spatial frequencies examined [34–36, 51, 53, 55, 58–60], while in others the greater decline corresponded to high spatial frequencies [34, 51, 61] and in still others the lower spatial frequencies were the most affected [49, 50, 52, 62]. Possible reasons for such discrepancies may lie in differences among samples and the CS test used [37]. Thus, the Regan chart, a low-contrast letter, and the Vistech VCTS 6500 are influenced by the VA value while the Pelli-Robson test and the Freiburg test are independent of VA [37]. In the present study, we use the CSV-1000 test which is based on the Pelli-Robson test and it can thus be assumed that it is not influenced by VA values.

In agreement with most studies on CS in AD, CS function was impaired in our cohort of early AD. However, the degree of involvement varied among spatial frequencies, the reduction being greater at the higher ones. Notably, in the studies reported in which CS was equally decreased in all spatial frequencies, AD patients were at a more advanced stage of the disease than were our MAD patients, as indicated by their lower MMSE values. There are only two studies with results consistent with ours, showing an increased decline at the higher spatial frequencies. However, both studies presented methodological differences with respect to ours. In the Neargarder et al. study [37], the results could have been influenced by the poorer VA of the patients, the test selected, and the great variability of MMSE (6–26) in the AD group. Meanwhile, the Gilmore et al. study [63] included only the participants who provided a valid response across all spatial frequencies, removing any patients who were not able to discriminate all checked spatial frequencies. Given these data, it seems that the 12- and 18-cpd spatial frequencies are the most affected in the early stages on the disease and thus as the disease progresses, all spatial frequencies become equally involved. According to our ROC values, the most sensitive spatial frequencies were 18 cpds (the highest), followed by the 12 cpds (85.7% and 83.2%, resp.); therefore, the highest spatial frequencies appear to have the highest diagnostic value in MAD neurodegeneration.

The fact that CS is impaired in AD patients in comparison with healthy elderly subjects has significant implications for cognitive abilities and daily function of AD patients [37], especially when the first spatial frequencies affected, according to our data, appear to be those corresponding to macular function.

### 4.3. Color Perception

When assessing color perception in our MAD patients, we found no significant differences in terms of color defects in comparison with age-matched control subjects. However, we found that our MAD patients made significantly more (72.62%) unspecific errors in the tritan (blue) axis than did the controls, which represented almost a threefold increase in the errors committed by the MAD group.

Unlike subjects with a loss of color vision due to focal cortical lesions, patients with AD rarely complain of color-vision deficits. Color testing in patients with AD is controversial because these patients have a deficit in naming colors and therefore might have trouble verbalizing the colors or nominating numbers and shapes that are being viewed. The color test used in the present study does not require naming, although it should be taken into account that test performance depends on the memory of the patients and therefore, for appropriate testing, the instructions need to be repeated by the examiner during test session, when necessary.

Some studies using the Farnsworth and Ishihara tests have found no color perception differences between patients with AD and the control group [58, 64, 65]. On the other hand, several studies using the City University Color Test found defects in the tritan axis and reported a correlation with the degree of dementia [30, 50, 66]. These data are consistent with the results reported by other authors using the Ishihara test [55] and the City University Color Test [67, 68]. By contrast, Pache et al., using the Ishihara test and the PV-16, found that unspecific errors not associated with a specific axis were more prevalent in AD patients compared to controls; however, this finding was not related to the severity of the disease [31]. This discrepancy among color-vision results in AD patients may be due to differences in the color test applied, so that comparisons of the results are difficult to interpret.

Salamone et al. postulated that the problem of color discrimination in patients with AD is not purely cognitive but seems to be related to damage in the structures responsible for the perception of color stimuli [32]. This statement is consistent with the evidence that extrastriate lesions may result in tritanomalous color deficits [50] and that the extrastriate cortex is severely affected in AD. The fact that no tritanomaly was found in our MAD patients but an increment of unspecific errors appeared along the tritan axis leads us to postulate that, in addition to the general loss of M cells and P cells that seems to be taking place in our patients (as mentioned above), the K pathway may also be involved, given that the blue-yellow spectrum is associated with this pathway [69]. Bistratified ganglion cells receive blue-on/yellow-off color-opponent excitation signals from the short-wavelength sensitive S cones and project this information to the koniocellular layers in the LGN [70].

### 4.4. Perception Digital Test (PDT)

The MAD patients of the present study made significantly more mistakes in PDT answers than did the age-matched control subjects. Given that no statistically significant differences in educational level were found among groups, the influence of this variable could be ruled out.

The part of the brain initially involved in AD is the temporal cortex, which is then followed by the temporoparietal association cortex [1, 71]. As a consequence, visual perception disorders are frequently associated with Alzheimer's neurodegeneration [71]. Before PDT development [46] visuoperceptual tests showed low sensitivity for detecting disorders in the early stages of the disease [72]. The development of more sensitive neuropsychological tests such as PDT has enabled the assessment of visual perception disorders in initial AD [46]. In view of the good predictive value (aROC = 0.758, *P* < 0.01) provided by the application of the PDT, we postulate PDT as a useful ancillary screening test for MAD.

### 4.5. aROC

To the best of our knowledge, no predictive (ROC) analysis is available for the diagnosis value of different visual psychophysical test in AD. A noteworthy finding of the present work was that the test with the greatest prognostic value in MAD patients was CS for 18-cpd and 12-cpd spatial frequencies (aROC 0.857 and 0.832, resp.), followed by VA (aROC 0.771), PDT (aROC 0.758), and unspecific errors in the blue region (aROC 0.714).

### 4.6. Pearson's Correlation

As mentioned above, our AD patients had a high mean MMSE value, the highest so far reported to the best of our knowledge. However, given that MMSE values ranged from 17 to 31, we investigated whether the MMSE value influences the test outcome. As a result, we found a significant correlation between MMSE values and PDT (*r* = 0.536), followed by the CS for 6-cpd (*r* = 0.516) and 18-cpd (*r* = 0.468) spatial frequencies, and VA (*r* = 0.457) and CS for 12-cpd (*r* = 0.451) and 3-cpd (*r* = 0.366) spatial frequencies. A negative correlation was also found between MMSE and the unspecific errors in the tritan region (*r* = −0329). All these findings showed that as the disease progresses, the answers to different tests used for assessing visual skills worsened.

Possible drawbacks of the study include the cross-sectional design, the subjective nature of the psychophysical testing (which depends on cognitive abilities), and the early stage of disease (mild cognitive impairment, which can be due to reasons other than AD). Another limitation could be the sample size. Gathering large samples is a difficult task when dealing with MAD patients. This was especially true in the present study in which the inclusion criteria were very demanding in order to recruit patients in early stages of AD who were also free of ocular pathology, the latter requisite limiting patient enrollment given the age range of the sample. Nevertheless, our sample size was comparable to that reported in the literature [36, 37, 40, 50, 51, 59].

## 5. Conclusions

In conclusion, our study demonstrates a deficit in visual perception in early stages of AD, manifested by reductions in VA, CS, and color discrimination. These psychophysical changes correlate with retinal morphological changes (decreased RNFL macular thickness as detected by OCT) detected in the same group of patients [9]. Furthermore, the observed changes in PDT suggest a visual integration deficit, possibly due to, at least in part, an incipient cortical dysfunction. The study of the predictive value of the tests analyzed herein showed that, in the most incipient AD grades, tests having the greatest predictive value are the CS, VA, unspecific errors in tritan region, and the PDT. The fact that no differences were detected between our MAD patients and age-matched control subjects in the dilated fundus examination highlights the importance of applying psychophysical tests in patients with MAD. Given the difficulty in gathering larger samples in AD, broader transverse as well as longitudinal studies would be useful to track changes in the psychophysical tests as the disease progresses.

## Figures and Tables

**Table 1:** Demographic and clinical data of the study groups.

	AD	Control	*P* value
	(*n* = 23)	(*n* = 28)	
Age^**∗**^	79.3 ± 4.6	72.3 ± 5.1	0.274
Gender			0.614
Men	9	9	
Female	14	19	
Race	Caucasian	Caucasian	
MMSE^**∗**^	23.3 ± 3.1	28.2 ± 1.9	0.001^††^
	Range (17–29)	Range (25–31)	
Educational level^**∗**^	1.43 ± 0.78	1.43 ± 0.79	0.950

^*∗*^Mean value ± SD; ^††^ *P* < 0.01 Mann-Whitney *U* test (AD: Alzheimer's disease; MMSE: Mini Mental State Examination; SD: standard deviation).

**Table 2:** Mean data and *P* value of the psychophysical tests.

Test	AD group	Control group	% difference	*P* value
Visual acuity (dec)^**∗**^	0.78 ± 0.19	0.95 ± 0.06	−17.89	0.001^††^
Contrast sensitivity^**∗**^				
3 cpds	1.39 ± 0.27	1.62 ± 0.19	−14.61	0.001^††^
6 cpds	1.58 ± 0.24	1.88 ± 0.22	−16.03	0.001^††^
12 cpds	1.12 ± 0.33	1.46 ± 0.20	−23.05	0.001^††^
18 cpds	0.61 ± 0.37	1.11 ± 0.27	−44.57	0.001^††^
Rue 28-hue^**∗**^				
Number of errors	7.41 ± 4.69	5.95 ± 4.06	24.54	0.18
Tritan unspecific errors	4.35 ± 2.40	2.52 ± 2.00	72.62	0.009^††^
PDT (sheet)^**∗**^				
Successful number of sheets	11.74 ± 2.39	13.79 ± 1.47	−14.87	0.01^†^

^**∗**^Mean value ± SD; ^†^ *P* < 0.05, ^††^ *P* < 0.01 Mann-Whitney *U* test (AD: Alzheimer's disease; cpds: cycles per degree; PDT: perception digital test).

**Table 3:** aROC analysis of psychophysical tests in mild Alzheimer's disease.

Psychophysical test	aROC	SD	*P* value
Visual Acuity	0.771	0.072	0.001^††^
Contrast sensitivity			
3 cpds	0.755	0.072	0.002^††^
6 cpds	0.808	0.061	0.001^††^
12 cpds	0.832	0.064	0.001^††^
18 cpds	0.857	0.055	0.001^††^
Rue 28-hue			
Number of errors	0.610	0.081	0.186
Tritan unspecific errors	0.714	0.074	0.01^†^
PDT (sheet)			
Successful number of sheets	0.758	0.068	0.002^††^

^†^ *P* < 0.05, ^††^ *P* < 0.01 Mann-Whitney *U* test (aROC: area under the receiver operating characteristic; AD: Alzheimer's disease; cpds: cycles per degree; PDT: perception digital test; SD: standard deviation).

**Table 4:** Pearson's correlation between psychophysical tests and Mini Mental State Examination.

Psychophysical test	*r*	*P* value
Visual acuity	0.457	0.001^††^
Contrast sensitivity		
3 cpds	0.366	0.011^††^
6 cpds	0.516	0.001^††^
12 cpds	0.451	0.001^††^
18 cpds	0.468	0.001^††^
Rue 28-hue		
Number of errors	−0.271	0.063
Tritan unspecific errors	−0.329	0.023^†^
PDT		
Successful number of sheets	0.536	0.001^††^

^†^ *P* < 0.05, ^††^ *P* < 0.01 Pearson's test (AD: Alzheimer's disease; cpds: cycles per degree; PDT: perception digital test).
